# LAPAROTOMIC RADIOFREQUENCY ABLATION OF PANCREATIC INSULINOMA

**DOI:** 10.1590/0102-6720202400026e1819

**Published:** 2024-08-30

**Authors:** Allan Rubens Zucolotto CANSI, Jhonatan de Souza VITOR, João Felipe da Silva LOPES, Rogério Dardengo GLÓRIA

**Affiliations:** 1 Hospital Evangélico de Cachoeiro de Itapemirim – Cachoeiro de Itapemirim (ES), Brazil.

**Keywords:** Insulinoma, Pancreas, Neoplasm, Insulin, Radio Waves, Insulinoma, Pâncreas, Neoplasia, Insulina, Ondas de Rádio

## Abstract

Insulinomas are rare neoplasms of the endocrine pancreas. Minimally invasive treatment options for insulinomas have gained prominence, replacing surgical resection due to its associated morbidity and mortality. Radiofrequency ablation (RFA) has emerged as a relevant treatment option. We present a case of a female patient with neuroglycopenic symptoms and severe hypoglycemic crises. The abdominal magnetic resonance imaging (MRI) showed a small nodular lesion in the pancreatic body. Laparotomy was performed, followed by RFA using a 15-mm active-tipped needle. No complications transpired, and no hypoglycemic episodes were observed during 12 months of follow-up.

## RESUMO

Os insulinomas são neoplasias raras do pâncreas endócrino. As opções de tratamento minimamente invasivas para os insulinomas têm ganhado destaque, substituindo a ressecção cirúrgica, devido à morbimortalidade associada. A ablação por radiofrequência (ARF) surgiu como uma opção de tratamento relevante. Apresentamos o caso de uma paciente do sexo feminino com sintomas neuroglicopênicos e crises hipoglicêmicas graves. A ressonância magnética (RM) abdominal revelou pequena lesão nodular no corpo pancreático. Foi realizada laparotomia seguida de ARF com agulha de ponta ativa de 15 mm. Nenhuma complicação ocorreu e nenhum episódio de hipoglicemia foi observado durante 12 meses de acompanhamento.

## INTRODUCTION

Insulinomas are rare pancreatic neuroendocrine tumors that secrete insulin. They have an incidence of approximately four per million inhabitants per year and are more commonly found in women aged under 45 years^1-4,6-9,11,14,16^. In about 90% of cases, they are small, solitary, and sporadic tumors (<2 cm)^4,9,10^. However, approximately 5–10% of insulinomas are associated with type 1 multiple endocrine neoplasia and are more likely to be multiple and malignant^1,5,8,12^.

In recent years, several minimally invasive treatment options have been used to treat insulinomas, increasingly replacing surgical resection, which, despite high cure rates, carries significant morbidity and mortality^1,4,5,8,11,12,14,17^. Radiofrequency ablation (RFA) of insulinomas is a relevant treatment option, with most reports indicating endoscopic ultrasound guidance^13,15,17^. Nonetheless, the limited availability of echo-endoscopy in Brazil emphasizes the need for the procedure to also be performed percutaneously, laparoscopically, or via laparotomy.

The objective is to describe the first case of pancreatic insulinoma treated with radiofrequency ablation via laparotomy.

## CASE REPORT

**Figure 1:**
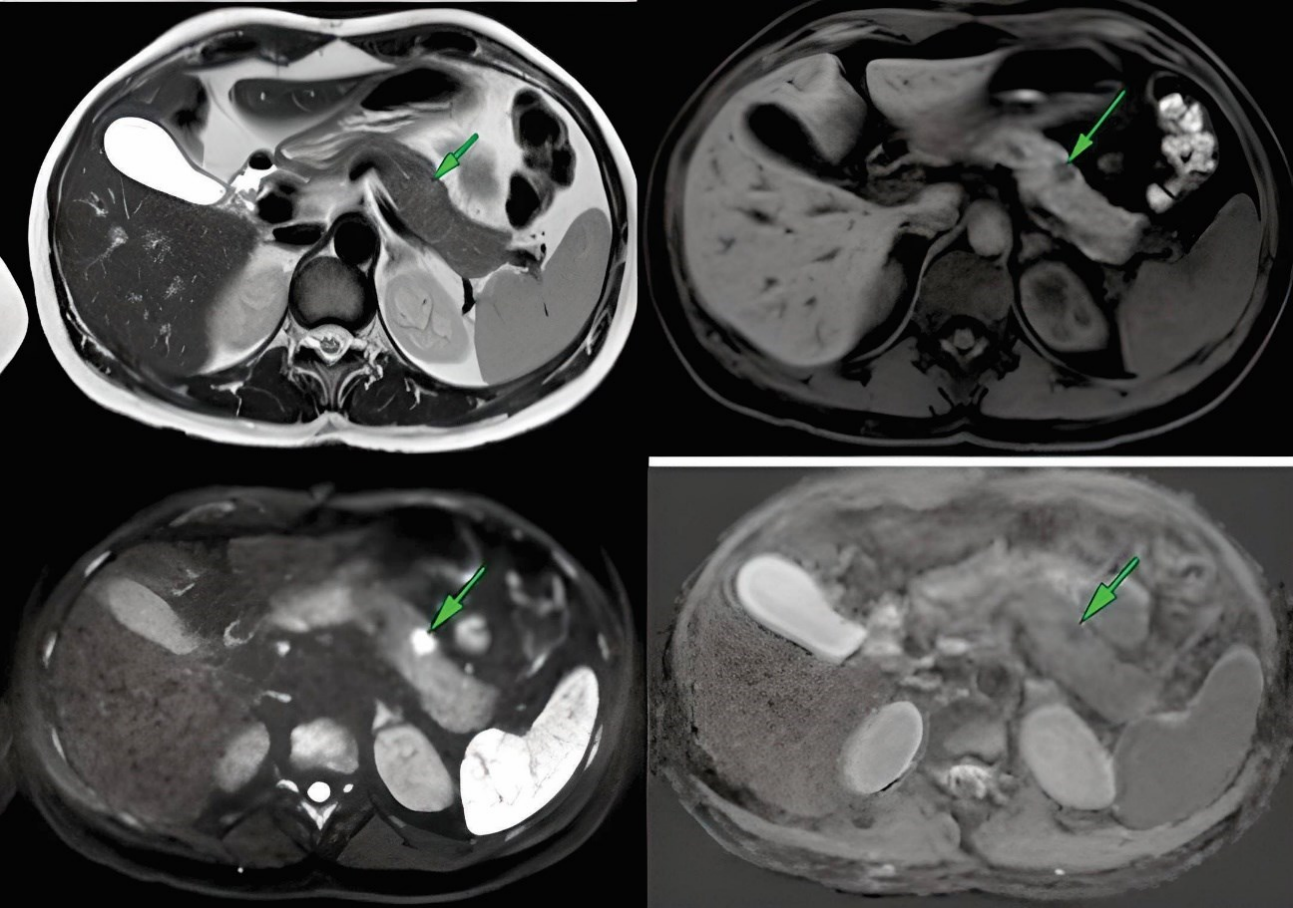
Preoperative magnetic resonance imaging.

A 37-year-old female patient, no prior comorbidities, presented with neuroglycopenic symptoms accompanied by hypoglycemia that had progressively worsened over three years. In the three months preceding definitive treatment, the patient experienced severe hypoglycemic crises multiple times a day, requiring interruptions in sleep to restore blood glucose levels. A follow-up with an endocrinologist raised the diagnostic hypothesis of insulinoma. An abdominal MRI (Figure 1) was conducted for further investigation, showing a small nodular lesion with hypersignal on T2 and hypointense signal on T1. The lesion presented heterogeneous and moderate contrast enhancement, along with significant restriction to the diffusibility of water. It was in the body of the pancreas, measuring 1.0x0.7 cm.

At the time of diagnosis, the patient presented an Hb1Ac level of 4.4% (reference >5.7%), fasting blood glucose of 27 mg/dL (reference <74–99 mg/dL), and neuroglycopenic symptoms that resolved upon glucose administration. These findings, collectively known as Whipple’s triad, were further supported by the identification of the lesion found on magnetic resonance imaging of the abdomen, confirming the diagnostic suspicion.

At our healthcare facility, the patient was presented with the option of surgical removal of the lesion, with a thorough explanation of the associated risks and benefits. Additionally, the alternative option offered was tumor ablation using radiofrequency. The patient received detailed information about both treatments’ modalities, through shared decision-making, opted for laparotomic RFA as the preferred course of treatment.

The patient was admitted to the operating room with a capillary blood glucose of 37 mg/dL, asymptomatic. After anesthetic induction, a 7-cm midline incision in the epigastrium was made by the pancreatic surgeon, and the access to the omental retrocavity was achieved. Employing intraoperative ultrasound with a high frequency linear transducer, the lesion of approximately 10 mm was identified in the pancreatic body, without direct contact with the Wirsung duct.

**Figure 2:**
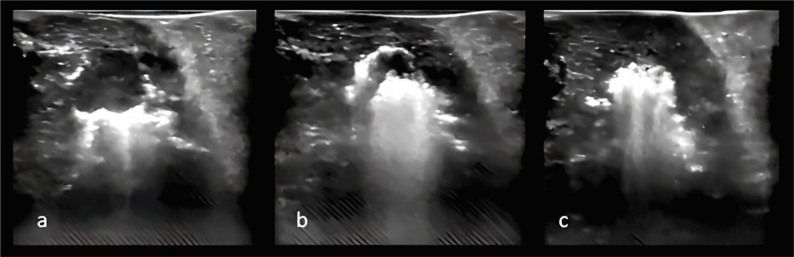
Intraoperative ultrasound (a, b and c).

Under ultrasound guidance, a biopsy of the lesion was obtained with an 18G needle, followed by the insertion of the RFA needle with a 15-mm active tip positioned at the center of the lesion. The initial protocol began with a power of 30W and a flow rate of 74 mL/h of 0.9% sodium chloride for 5 minutes, during which complete ablation did not occur. A subsequent cycle was initiated with a 9-minute protocol, employing a power of 70W and a 0.9% sodium chloride solution flow at 130 mL/h. This resulted in a significant alteration throughout the entire architecture of the tumor as seen on real-time intraoperative ultrasound (Figure 2).

At the end of the procedure, which lasted a total of 70 minutes, no bleeding, nor intraoperative complications, were observed.

In the immediate postoperative period, the patient’s capillary blood glucose were 189 mg/dL, 163 mg/dL, and 149 mg/dL in the first 18 hours. She did not present any neuroglycopenic symptoms, being discharged from the hospital on the first postoperative day. Since then, no other episode of hypoglycemia was seen over the following 20 months.

**Figure 3:**
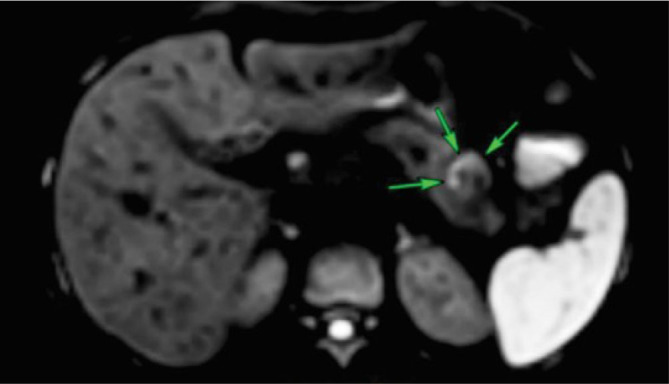
Magnetic resonance imaging six months postoperatively.

Laboratory tests for control, conducted six months after the procedure, demonstrated fasting glucose at 86 mg/dL, amylase at 54 U/L, C-peptide at 1.80 ng/mL, Hb1Ac at 4.8%, and insulin at 7.7 micro-IU/mL. Upper abdominal MRI, performed six months after RFA, displayed a RFA area measuring 1.8x1.7 cm in the anterior part of the body of the pancreas, where the lesion was found before (Figure 3).

## DISCUSSION

This report describes a young patient with a solitary insulinoma located in the pancreatic body, marked by frequent hypoglycemia episodes and a worsening clinical course.

Insulinomas are often misdiagnosed as neurological or psychiatric disorders, resulting in delayed diagnosis. In a retrospective study, 64% of patients presenting neuropsychiatric symptoms alongside insulinoma were not accurately diagnosed within 12 months after their initial consultation^6^.

In recent years, several new treatment options have emerged for managing insulinomas, progressively replacing surgical removal due to its substantial morbidity despite high cure rates^4,8,10,11^. Among the available alternatives are RFA, high-frequency focused ultrasound ablation intensity, microwave ablation, ethanol ablation, and irreversible electroporation^4,9^.

Different approaches have been documented for insulinoma treatment using RFA, including laparoscopic, endoscopic, and percutaneous methods. However, this particular case marks the first utilization of a laparotomy approach. The choice of treatment considered the patient’s unique circumstances and preferences, along with the equipment availability in the facility. The percutaneous approach, though less invasive, faced limitations due to challenges associated with puncturing the pancreas, which is deeply situated in the retroperitoneum and positioned anteriorly to organs such as the stomach and colon, with the spine posteriorly.

Endoscopic ultrasound-assisted RFA (EUS) is the preferred approach when a better access route to the transgastric or transduodenal tumor lesion is required. Yet, EUS has limited availability, requires experienced professionals, and carries the risk of gastrointestinal tract damage. Laparoscopic RFA is another viable option, but its utilization was hindered in our case due to the unavailability of a laparoscopic transducer ultrasound, crucial for precise tumor delimitation, and a lack of experienced professionals in this specific procedure within our service.

## CONCLUSIONS

Reports such as ours hold significant value in demonstrating the viability of innovative and less invasive techniques for treating insulinomas in public healthcare facilities or hospitals that are not affiliated with major medical centers. This ensures a broader segment of the population to have access to promising treatments with superior outcomes.
